# Sustainable Composites from Waste Polypropylene Added with Thermoset Composite Waste or Recovered Carbon Fibres

**DOI:** 10.3390/polym16202922

**Published:** 2024-10-18

**Authors:** Ehsan Zolfaghari, Giulia Infurna, Sabina Alessi, Clelia Dispenza, Nadka Tz. Dintcheva

**Affiliations:** Dipartimento di Ingegneria, Università degli Studi di Palermo, Viale delle Scienze, ed. 6, 90128 Palermo, Italy; giulia.infurna@unipa.it (G.I.); sabina.alessi@unipa.it (S.A.); clelia.dispenza@unipa.it (C.D.)

**Keywords:** waste polypropylene, carbon-fibre-reinforced epoxy composite scrap, recovered carbon fibres

## Abstract

In order to limit the ever-increasing consumption of new resources for material formulations, regulations and legislation require us to move from a linear to a circular economy and to find efficient ways to recycle, reuse and recover materials. Taking into account the principles of material circularity and waste reuse, this research study aims to produce thermoplastic composites using two types of industrial waste from neighbouring companies, namely waste polypropylene (wPP) from household production and carbon-fibre-reinforced epoxy composite scrap from a pultrusion company. The industrial scrap of the carbon-fibre-reinforced epoxy composites was either machined/ground to powder (pCFRC) and used directly as a reinforcement agent or subjected to a chemical digestion process to recover the carbon fibres (rCFs). Both pCFRC and rCF, at different weight ratios, were melt-blended with wPP. Prior to melt blending, both pCFRC and rCF were analysed for morphology by scanning electron microscopy (SEM). The pCFRC powder contains epoxy resin fragments with spherical to ellipsoidal shape and carbon fibre fragments. The rCFs are clean from the matrix, but they are slightly thicker and corrugated after the matrix digestion. Further, the morphologies of wPP/pCFRC and wPP/rCF were also investigated by SEM, while the thermal behaviour, i.e., transitions and changes in crystallinity, and thermal resistance were evaluated by differential scanning calorimetry (DSC) and thermogravimetric analysis (TGA), respectively. The strength of the interaction between the filler (i.e., pCFRC or rCF) and the wPP matrix and the processability of these composites were assessed by rheological studies. Finally, the mechanical properties of the systems were characterised by tensile tests, and as found, both pCFRC and rCF exert reinforcement effects, although better results were obtained using rCF. The wPP/pCFRC results are more heterogeneous than those of the wPP/rCF due to the presence of epoxy and carbon fibre fragments, and this heterogeneity could be considered responsible for the mechanical behaviour. Further, the presence of both pCFRC and rCF leads to a restriction of polymer chain mobility, which leads to an overall reduction in ductility. All the results obtained suggest that both pCFRC and rCF are good candidates as reinforcing fillers for wPP and that these complex systems could potentially be processed by injection or compression moulding.

## 1. Introduction

Polypropylene (PP) is one of the most widely used thermoplastic polymers due to its wide range of essential properties, which are favourable for various end-use applications and long-lasting applications when PP is combined with suitable additives. It is well known that PP is a versatile material with a high melting point and excellent properties such as chemical resistance, low density, high tensile strength and good fatigue resistance [1,2,3]. According to statistical data from Polaris Market Research, PP consumption is growing steadily and is expected to increase at a compound annual growth rate of 4.7% by 2030 [4]. The growth can be attributed to the increase in PP consumption in the automotive, packaging, household item, building and construction sectors. The automotive sector has set itself the goal of producing lightweight vehicles to reduce fuel consumption, and polypropylene can be an excellent option for achieving this goal due to its mechanical properties and chemical resistance, which are even more interesting when recycled PP from industrial scrap and/or post-consumer waste is used. In addition, favourable properties of PP, such as solvent resistance, chemical inertness and thermal resistance, mean that its use in the production of packaging and household items is steadily increasing. Interestingly, though, although the consumption of PP is considerable, its recycling rate in terms of material recovery is insufficient [5,6,7,8]. This is mainly due to a lack of adequate strategies to handle the recycling process [9].

Although PP has many good properties, for some end-use applications it needs to be combined with appropriate additives such as reinforcing fillers, impact modifiers and stabilisers to further improve its mechanical properties, impact resistance and durability [1,2,3]. This is even more true when considering the use of recycled PP, which comes from production scrap and/or post-consumer waste [9,10,11,12,13]. The reinforcing filler is frequently a fibrous material. The role of the fibres is to provide the strength of the composite, while the PP matrix holds the fibres in specific positions and orientations, providing a specific shape and protection from environmental damage [1,2,3,8,14,15,16,17,18,19,20,21,22,23,24,25,26]. There are two main categories of fibres: natural and synthetic. Synthetic-fibre-reinforced thermoplastic composites have better mechanical properties, durability and moisture resistance properties compared to natural-fibre-reinforced ones [8,14]. The most commonly used synthetic fibres in composites are glass fibres [15,16,23] and carbon fibres [17,18,19,20]. In particular, carbon-fibre-reinforced polypropylene (CFRPP) composites combine the superior mechanical properties of carbon fibres with the versatility of PP to formulate composites with improved strength, stiffness and durability [24].

Interestingly, in recent years there have been several studies on fibre-reinforced PP composites that have looked at the introduction of other reinforcing additives in combination with to carbon fibres achieve further property improvements [19,20,21,22,23,24,25,26]. For example, Matsumoto and Takemura (2022) documented that the addition of cellulose nanofibres (CNFs) to carbon fibre surfaces can promote the trans-crystallisation of PP in the composites, increasing the flexural yield strength and modulus by up to 30% and 63%, respectively [21]. Furthermore, Franciszczak et al. (2018) investigated the combination of cellulose and e-glass fibres in polypropylene-based composites [23]. They found significant improvements in the mechanical properties, especially the flexural strength of the composite, by reducing the void content and fibre damage because of increasing the matrix-to-filler stress transfer. Interestingly, the authors concluded that the addition of an extra volume of a second reinforcement with a lower Young’s modulus increases to some extent the Young’s modulus of composites, although there was an adverse effect on the strength of composites due to the inefficient stress transfer from the matrix at the deformation of the composites that collaterally caused the length reduction in the primary fibre during processing [23].

In another study, He et al. (2022) manufactured conductive PP/multiwalled carbon nanotube (MWCNT) composites using microcellular injection moulding, which resulted in major improvements in mechanical properties, dielectric properties and electromagnetic shielding. As documented, the introduction of polytetrafluoroethylene (PTFE) in the PP/MWCNTS composites provided a better fibre–matrix interaction and improved the composite elongation at break and overall performance [25]. Tsioptsias et al. (2022) investigated the use of micro-fillers, such as micro-talc, ultrafine talc, wollastonite, attapulgite and single-wall carbon nanotubes, in PP composites and their impact on the applicability and final performance of the composites. In addition, there are suitable antioxidants and compatibilizers, such as PP grafted with maleic anhydride, that are also used to further improve the properties and performance of isotactic PP-based composites. The investigation revealed that needle-like fillers, e.g., wollastonite and single-walled carbon nanotubes, because of their high aspect ratio, align during the drawing process and the latter leads to better stress distribution and enhanced mechanical properties [26].

Other important studies report on the effects of the introduction of nanofillers of various compositions, such as clay, montmorillonite, calcium carbonate, carbon nanotubes, silica, zinc oxide and graphene, in recycled PP to formulate second-life materials with improved performance, i.e., mechanical, thermal, rheological, barrier and electrical properties [27,28,29,30,31,32].

As is known, carbon-fibre-reinforced epoxy resins have attracted great interest from an industrial point of view, also for the formulation of products that can replace metal alloy components for energy recovery in the automotive and construction sectors [33,34]. The main production technique for the formulation of carbon-fibre-reinforced epoxy-based resins is pultrusion technology, consisting of the impregnation of the fibre and curing of the resins, up to the formulation of final objects/items having excellent mechanical performance. However, a recent review by Morici et al. [35] on the recycling of thermosets and thermoset composites highlights the pathways described in the literature. To the best of our knowledge, the reinforcement of recycled polypropylene with fibres also recovered from industrial waste has not yet been addressed in the scientific literature. However, it is well known that thermoset composite waste is a serious problem in most countries due to its environmental and health risks. Efforts are needed to give a second life to this composite waste, thereby reducing its quantity and limiting the consumption of new resources.

Taking into account the principles of material circularity, in this work sustainable composites were formulated using two types of industrial waste from neighbouring companies, namely waste polypropylene (wPP) from household production and carbon-fibre-reinforced epoxy composite scrap from a pultrusion company. The carbon-fibre-reinforced epoxy composite scrap was either pulverised (pCFRC) or subjected to a chemical digestion process to recover the carbon fibres (rCF). The wPP was then mixed by melt blending with pCFRP at 1 wt%, 10 wt% and 20 wt% and with rCF at 10 wt% and 20 wt% and the formulated composites were subjected to rheological, mechanical, morphological and thermal analyses. The results obtained suggest that “neat” wPP exhibits fragile behaviour and can be successfully reinforced by the addition of both pCFRC and rCF.

## 2. Materials and Methods

### 2.1. Materials

The waste polypropylene (wPP) was supplied by Euroscope (Italy) from household production waste and was used as received from the company in the formulation of the composites. Powder waste from the cutting of carbon-fibre-reinforced epoxy resins (pCFRP) was kindly supplied by K-Composite s.r.l (Italy) and used as received in the formulation of some composites. Reclaimed carbon fibres (rCF) were obtained through a standard solvolysis process [34,35,36] from carbon-fibre-reinforced epoxy resin waste, kindly supplied by K-Composite s.r.l. Virgin carbon fibres (vCF) were also kindly provided by K-Composite s.r.l.

### 2.2. Methods for Composite Formulation and Characterizations

#### 2.2.1. Composites Formulation by Melt Micing

wPP/pCFRC and wPP/rCF formulations were processed by melt blending using a Brabender mixer (cams in counter-rotating mode) at the temperature of T = 190 °C for 5 min, i.e., until the achievement of constant torque values. To formulate wPP/pCFRC and wPP/rCF composites, the pCFRP was introduced in wPP at 1%wt, 10%wt and 20%wt, while the rCF was introduced at 10%wt and 20%wt, respectively. For comparison, wPP was subjected to the same processing conditions. Sheets, having a thickness of ca. 0.3–0.4 mm, requested for the characterizations were obtained by the hot compression moulding process using a Carver press at the temperature of 190 °C. The compositions of all considered samples are reported in Table 1.

#### 2.2.2. Scanning Electron Microscopy (SEM)

The morphologies of the pCFRC-, rCF- and wPP-based composites’ fractured surfaces were investigated using a Scanning Electron Microscope (SEM FEI Quanta 200 FEG) equipped with an X-ray energy-dispersive spectrometer (EDS) at 20.00 kV. Prior to the examination, wPP/pCFRC and wPP/rCF samples were fractured in liquid nitrogen, placed onto a conductive stub, and then gold-sputtered to avoid electrostatic charging effects.

#### 2.2.3. Differential Scanning Calorimetry (DSC)

DSC analysis was performed with Labsys evo TG-DSC SETARAM under nitrogen flow in the temperature interval 20 °C–250 °C, performing three consecutive ramps (first heating, first cooling and second heating). The heating rate was 10 °C/min. The DSC analysis was carried out using an aluminium pan and lid with a volume of 40 mL in a nitrogen atmosphere. The weight of each sample was approximately 10 mg. Data were acquired and analysed using Pyrex Software v1.4, provided by the manufacturer.

#### 2.2.4. Thermo-Gravimetric Analysis (TGA)

TGA was conducted using a Labsys evo TG-DSC SETARAM under an air atmosphere, covering a temperature range from 25 °C to 600 °C and a heating ramp of 10 °C/min. The TGA was carried out using an alumina pan under an air atmosphere. The weight of each sample was approximately 10 mg. Data were acquired and analysed using Calisto Software v1.36 provided by the manufacturer.

#### 2.2.5. Spectroscopy Analysis

A Fourier-Transform Infrared Spectrometer (Spectrum One, Perkin Elmer, Shelton, CT, USA) was used in Attenuated Total Reflectance (ATR-FTIR) mode to record ATR-FTIR spectra considering 16 scans at a resolution of 4 cm^−1^ in the range 4000–500 cm^−1^. The active element for acquiring the ATR-FTIR spectra was a diamond.

#### 2.2.6. Rheology Analysis

Rheological tests were performed using a stress-controlled rheometer (ARES G-2) in parallel-plate geometry (plate diameter 25 mm). The complex viscosity (η*), storage (G′) and loss (G″) moduli were measured under frequency scans from ω = 10^−1^ to 100 rad/s at T = 190 °C. The strain amplitude was γ = 5%, which preliminary strain sweep experiments proved to be low enough to be in the linear viscoelastic regime.

#### 2.2.7. Tensile Test

Monoaxial tensile tests were conducted with Instron 3360 on rectangular samples (10 mm width, 0.3–0.4 mm thickness and with a gauge length of 30 mm) produced by compression moulding. The crosshead speed was 1 mm/min. Tests were performed at 23 °C and 50% humidity. Ten samples were tested for each material and the deviations were calculated.

## 3. Results and Discussion

### 3.1. Morphological Analysis of pCFRC and rCF

The main objective of this study is to assess whether two types of industrial waste can be combined to produce a novel composite material with application potential. The polypropylene matrix was obtained from the production scraps of household goods. The carbon fibres were recovered from scrap carbon-reinforced composites consisting of off-spec parts from a pultrusion company. The recovery of the fibres was performed following a solvolysis process described in the literature [34,35,36]. As an alternative reinforcement, carbon fibre composite powder was also considered. The powder is another type of waste of the pultrusion company that is produced by cutting a variety of pultruded composites to the desired length. The powder was used after sieving on a 0.25 mm mesh.

**Figure 1:**
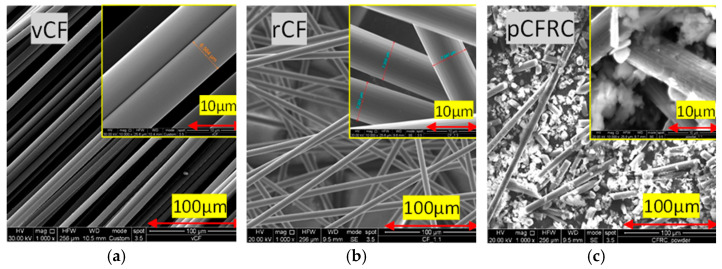
SEM images of the reinforcing additives at different magnifications: (**a**) virgin carbon fibre (vCF), (**b**) recovered fibres after matrix digestion (rCFs) and (**c**) powder (pCFRC).

In order to carry out an accurate visual inspection of the reinforcing additives under consideration, SEM observations were carried out and Figure 1 shows the most representative micrographs. For comparison, the SEM observations of the virgin carbon fibres used for the industrial production of the carbon-reinforced epoxy resin composites used in this study are also shown. It is worth noting that vCF appears smooth and without surface defects due to the presence of sizing; see Figure 1a. The rCFs are clean from the matrix, but slightly thicker and corrugated after matrix digestion, perhaps because part of the sizing was removed during digestion and the residual part was swollen by the solvent; see Figure 1b. Based on various observations, the length of the rCF was estimated to be between 10 mm and 15 mm; this size is controlled by the size and shape of the composite parts that were subjected to solvolysis and fibre orientation in the laminate. The pCFRC powder contains epoxy resin fragments with spherical to ellipsoidal shapes, partially attached to the fibres, and carbon fibre fragments with lengths ranging from a few microns to hundreds of microns; see Figure 1c.

### 3.2. Thermal Analysis of wPP/pCFRC and wPP/rCF Composites

To formulate sustainable composites, waste polypropylene (wPP) from production scrap was blended by melt mixing with powder (pCFRC) or recovered carbon fibres (rCFs), as summarised in Table 1. The produced composites were subjected to calorimetric, thermogravimetric, spectroscopic, morphological, rheological and mechanical analyses.

**Figure 2:**
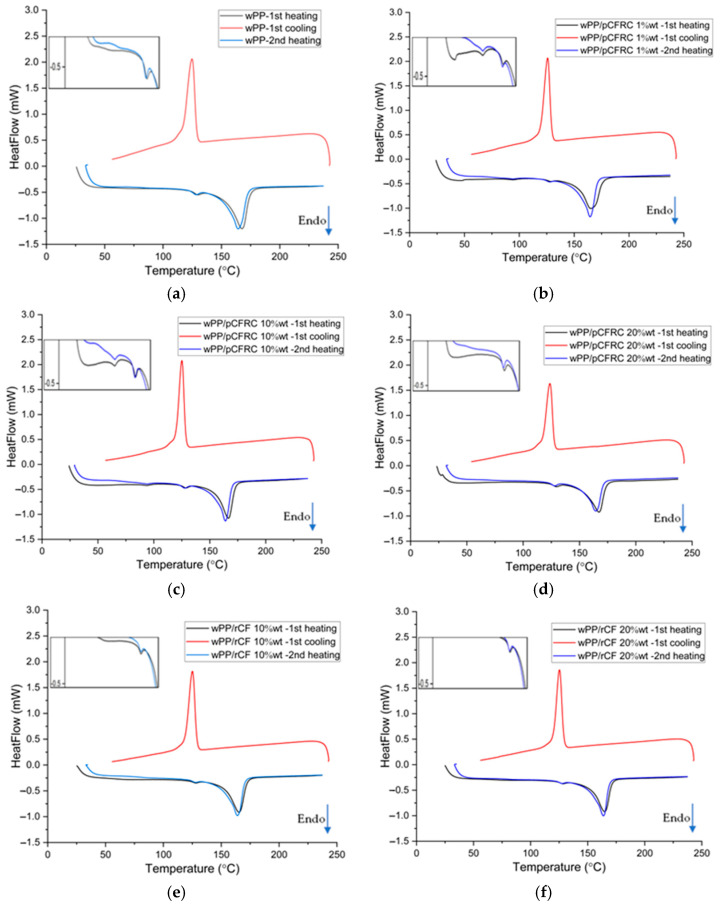
DSC traces of first and second heating (endotherm-down) and first cooling (exotherm-up) cycles for different composite formulations: (**a**) wPP alone, (**b**–**d**) wPP/pCFRC at varying concentrations of pCFRC and (**e**,**f**) wPP/rCF composites at two different rCF ratios.

**Scheme 1:**
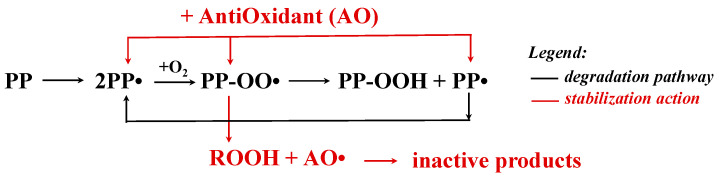
Overall degradation and stabilisation pathways of PP under thermo-mechanical stress, which illustrates the formation of free radicals in the absence of antioxidants, leading to a degradation of products (readapted from [37]).

Figure 2a–f show the first and second heating scans and the first cooling scan of unfilled wPP, wPP/pCFRC and wPP/rCF composites, and further, Table 2 reports all data from the calorimetric analysis. As is noticeable in Figure 2a, which refers to only wPP, the first heating scan shows a small initial melting peak at ca. 129.7 °C and a more pronounced melting peak at ca. 167.6 °C. Similarly, the second heating scan shows two melting peaks resulting at slightly lower temperatures, i.e., at ca. 128.5 °C and ca. 164.4 °C, suggesting the presence of two different crystal structures and/or a wide range of crystal sizes. The cooling scan of wPP shows a single sharp crystallisation peak at ca. 124.7 °C. Interestingly, the DSC traces of all wPP/pCFRC and wPP/rCF composites show two-step heating and single-step cooling scans, see Figure 2b–f, and the values of the melting and crystallisation temperatures of all wPP-based composites are very close to those of unfilled wPP; see the data in Table 1. These results suggest that both pCFRC and rCF are unable to exert a nucleating effect on wPP or significantly influence the phase transition temperatures of wPP. On the contrary, the presence of both pCFRC and rCF in wPP causes a decrease the melting and crystallisation enthalpies with respect to the ones of “neat” wPP of ca. 20%, irrespective of filler type and content. The fact that we do not observe a clear concentration dependence may suggest that the reduction in melting and crystallisation enthalpies is mainly attributable to possible changes in PP structure during the processing of the composite blend rather than to a hindrance effect of the fillers on the wPP crystallisation process. It is well known that PP is extremely susceptible to thermo-mechanical degradation and always requires the addition of stabilisers and other additives that prevent deterioration in its properties during processing. In this research, during reprocessing, no additives were added, and wPP was subjected to further thermo-mechanical degradation with the formation of free radicals, which can evolve into oxygen-containing groups when oxygen is available; see Scheme 1. According to the literature, the change in composition due to the formation of free radicals and oxygen-containing groups could lead to a deterioration in the overall material properties and performance, also because of a change in crystallinity [37].

**Figure 3:**
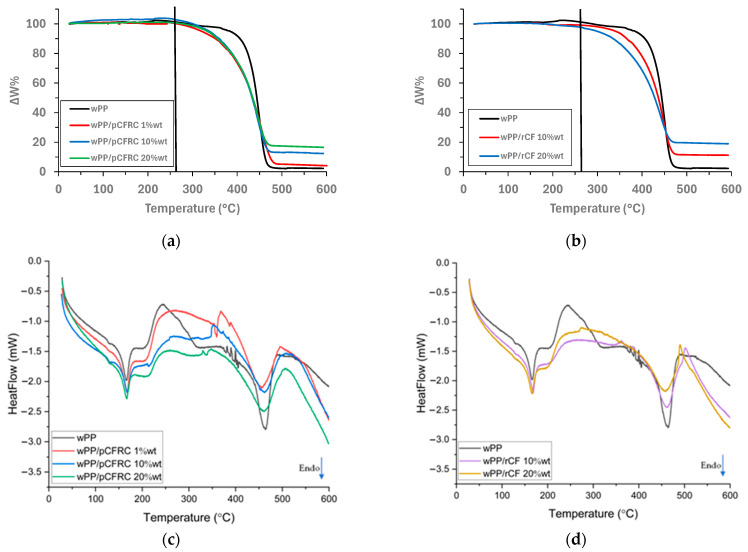
(**a**,**b**) TGA traces and (**c**,**d**) heat flow traces of wPP/pCFRC and wPP/rCF composites (endotherm-down); for comparison, the traces of wPP are also shown.

To investigate the thermal resistance of the wPP/pCFRC and wPP/rCF composites, TGA of unfilled wPP and all wPP-based composites was carried out. In Figure 3a–d, TGA traces (a,b) and corresponding heat flow traces (c,d) in the temperature range from 25 °C to 600 °C are shown. Further, Table 3 shows the data revealed by the trends in Figure 3a,b. As expected, the unfilled wPP shows no changes in weight up to approximately 400 °C and a well-pronounced single-step weight loss between 400 °C and 500 °C, paralleled by an endothermic peak. The residual mass of wPP at 600 °C is ca. 2.3%, probably due to the presence of carbonaceous residues formed during the heating scan at 10 °C/min due to molecular oxygen transfer resistance. The onset of weight loss for the wPP/pCFRC composites occurs at ca. 250 °C, irrespective of pCFRC content, well before the T_onset_ of wPP. The decrease in thermal degradation resistance could be attributed to the observed decrease in crystallinity as a result of the processing of the composite blend by melt mixing. The presence of higher residual fractions at 600 °C can be explained by the presence of carbon fibre fragments. A slightly different behaviour is shown by the rCF/wPP systems, where the onset of the main degradation process also depends on the rCF content, being at ca. 300 °C for wPP/rCF 10%wt and ca. 250 °C for wPP/rCF 10%wt. Additionally, in Figure 3c,d, heat flow curves as function of the temperature, from 20 °C up to 600 °C, are plotted. The endothermic peak at the lowest temperature (about 165 °C) is due to wPP melting. After that, we observe alternation in the endothermic and exothermic peaks, especially in the wPP/pCFRC composites, that suggests the occurrence of different thermo-oxidative degradation phenomena, involving the epoxy resin particles and wPP, with volatile degradation product formation and evolution.

### 3.3. Rheological Analysis of wPP/pCFRC and wPP/rCF Composites

**Figure 4:**
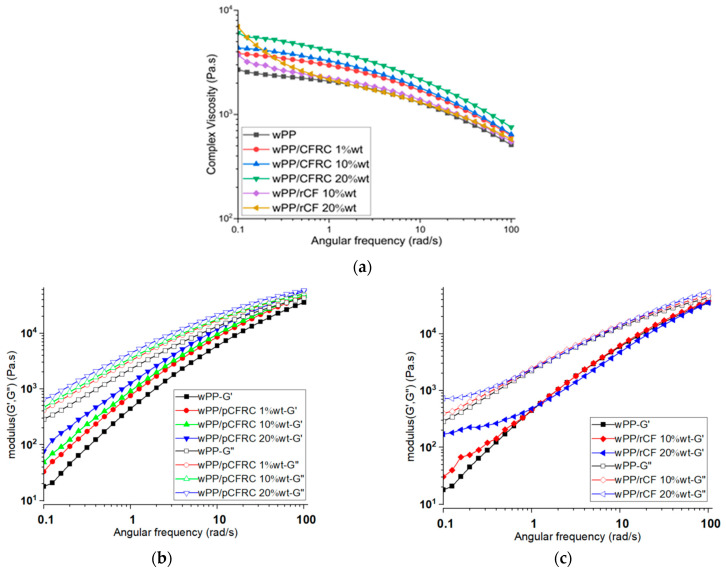
Rheological data for composite materials: (**a**) complex viscosity vs. angular frequency for all samples, (**b**) G′ and G″ moduli vs. angular frequency for wPP/pCFRC composites at different concentrations of pCFRC and (**c**) G′ and G″ moduli vs. angular frequency for wPP/rCF composites at various rCF ratios.

Figure 4a–c shows the trends of viscosity (η*) and storage (G′) and loss (G″) moduli for unfilled wPP melt and the wPP/pCFRC and wPP/rCF composite melts as a function of the angular frequency. As expected, wPP shows G″ > G′ (liquid-like behaviour) and a complex viscosity that is little to almost not affected by frequency at low frequencies (Newtonian behaviour in the rubbery plateau region) and decreases with frequency at high frequencies (shear thinning behaviour) [38,39]. Conversely, wPP/pCFRC composites show a shear thinning behaviour in the whole frequency range. This is probably because the filler interferes with the formation on an extended transient network where the crosslinking points are represented by chain entanglements. Interestingly, the higher the filler content, the higher the complex viscosity and storage modulus at all frequencies, probably as a result of an increased contact area between pCFRC and the polypropylene matrix. The increases in the non-linear effects on viscosity and moduli with concentration for wPP/pCFRC suggest that filler aggregation may occur at higher filler contents. It is likely that the interaction at the interface between wPP and fibre is facilitated by the presence of epoxy fragments and/or the presence of carbon fibre sizing. For the wPP/rCF composite melts, at lower frequencies the complex viscosity increases with the rCF content, due to the reinforcing effect of the interconnected randomly oriented fibre networks. During the test, the shear alignment of the rigid fibres causes the viscosity to decrease and to become equal to that of unfilled wPP. The reinforcing effect, present only at low angular frequencies, is also supported by the higher storage modulus [40,41,42].

### 3.4. Morphological Analysis of wPP/pCFRC and wPP/rCF Composites

**Figure 5:**
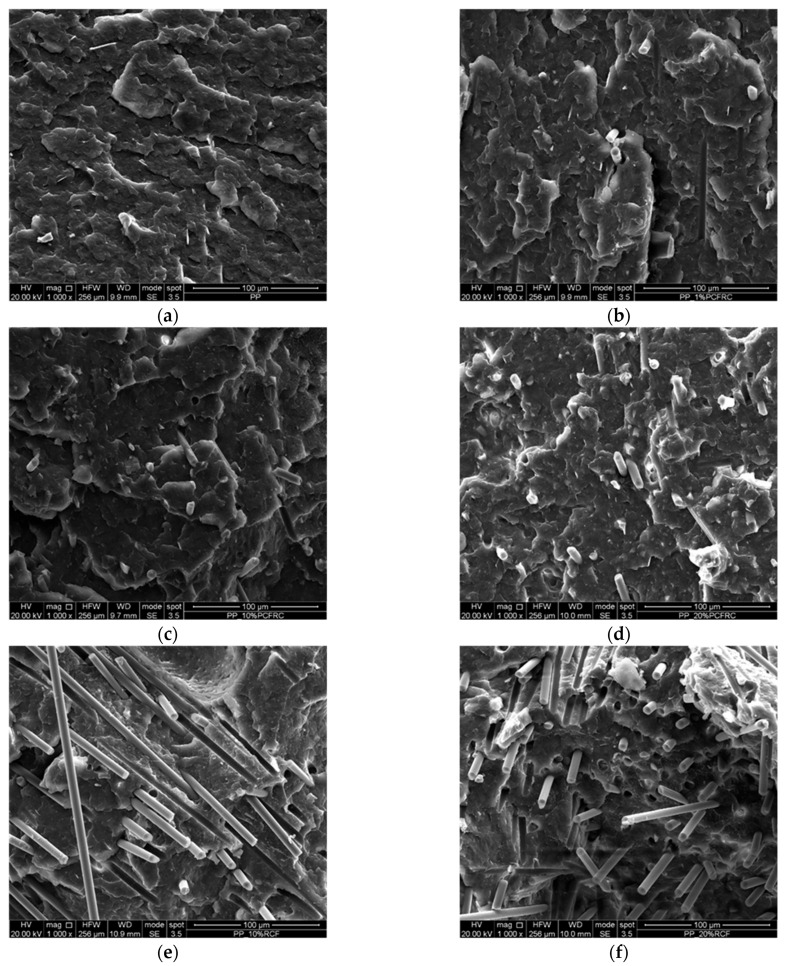
SEM images at identical magnifications: (**a**) wPP alone, (**b**–**d**) wPP/pCFRC at different concentrations of pCFRC and (**e**,**f**) wPP/rCF composites at various rCF ratios.

Detailed SEM observations of the wPP, wPP/pCFRC and wPP/rCF composites were made considering the samples used for mechanical testing and looking at their fracture surfaces. Representative images are shown in Figure 5a–f. It is clearly visible that all the samples, e.g., unfilled wPP, wPP/rCFRC and wPP/rCF composites, show a rough morphology. The morphology of the fracture surfaces of all composite systems shows well-distributed fibres in the composites, and no signs of coarse aggregation of epoxy resin particles. The morphology of the wPP matrix it is not significantly influenced by the presence of the reinforcing elements either.

As expected, the wPP/pCFRC samples show short carbon fibres; their presence increases with increasing filler concentration; see Figure 5b–d. These samples show good adhesion between the matrix and the pCFRC reinforcement, without obvious gaps at the matrix–filler interface and without many voids caused by fibre pool-out. Because of the similarity between wPP and epoxy fragments, no evidence of their presence can be derived from microscopic investigations.

The wPP/rCF samples show some fibres emerging from the fracture surfaces, some imprints and voids left by fibres that have been pulled away and several fibres that have broken off at the fracture surface; see Figure 5e,f. The adhesion between the wPP and rCF is not as good as for wPP/pCFRC composites, and this is probably due to the partial removal of fibre sizing during solvolysis. Small pores are also visible, probably due to the evaporation during the melt mixing of residual solvent during the fibre recovery process.

### 3.5. Spectroscopic Analysis of wPP/pCFRC and wPP/rCF Composites

**Figure 6:**
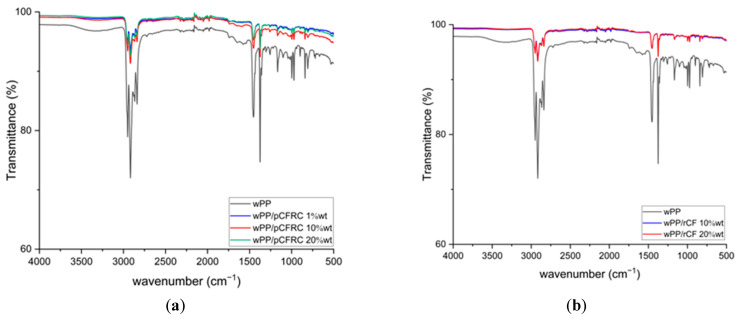
ATR-FTIR spectra of wPP and composite materials: (**a**) wPP/pCFRC and (**b**) wPP/rCF composites.

ATR-FTIR spectra of unfilled wPP and all wPP-based composites are shown in Figure 6a,b. As can be seen, there are no significant differences, in terms of new peaks or peak shifts, among the spectra due to the presence of pCFRC or rCF, regardless of their content.

### 3.6. Tensile Test of wPP/pCFRC and wPP/rCF Composites

**Figure 7:**
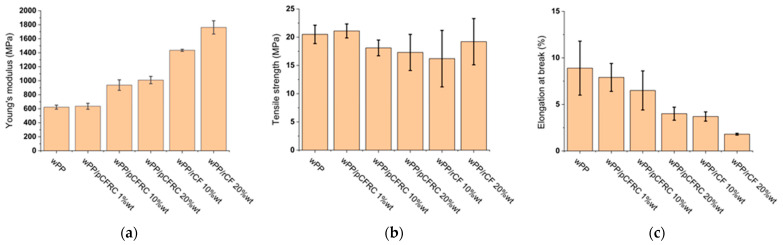
Mechanical properties of composite materials: (**a**) Young’s modulus, E; (**b**) tensile strength, TS; and (**c**) elongation at break, EB, of wPP and all wPP composites at different reinforcement concentrations.

To investigate the effect of both pCFRC and rCF on the mechanical behaviour, a mono-axial tensile test was carried out and the main mechanical properties obtained, Young’s modulus (E), tensile strength (TS) and elongation at break (EB), are shown in Figure 7a–c, respectively. It can be seen that the addition of pCFRC leads to a significant increase in the Young’s modulus values, and even more so the addition of rCF; see Figure 7a. Young’s modulus of wPP/rCF 20%wt increases more than three times compared to unfilled wPP, highlighting an excellent reinforcement effect in the solid state.

Tensile strength is not significantly affected by the presence of both pCFRc and rCF, regardless of filler type and content; see Figure 7b. The maximum reductions achieved in tensile strength are about 15% for wPP/pCFRC 10%wt and for wPP/pCFRC 20%wt, compared to wPP. This cannot be considered a drastic decrease, taking into account the inhomogeneous nature of the samples. Unfilled wPP shows low ductility; its elongation at break is less than 10%. As expected, all wPP-based composites show reduced ductility due to the restriction of polymer chain mobility by the presence of the stiff carbon fibres, which makes the composites less capable of elongation under stress. The mechanical behaviour obtained suggests that the wPP/pCFRC and wPP/rCF systems can be considered as good candidates for the production of rigid parts and items, by compression and/or injection moulding. Therefore, according to morphology analysis, the wPP/pCRFC show intrinsic heterogeneity due to the presence of both epoxy and carbon fibre fragments, and this could be considered responsible for the mechanical behaviour. The wPP/rCF show higher strength and, as expected, significantly reduced properties of break, also because the sizing was partially reduced during the matrix digestion.

## 4. Conclusions

Taking into account the principles of increasing material circularity and waste reuse, this study aims to formulate sustainable composite materials using waste polypropylene from production scrap (wPP) with machined-to-powder waste of carbon-fibre-reinforced composites (pCFRC) and recovered carbon fibres (rCFs).

The calorimetric analysis suggests that the melting and crystallisation temperatures of wPP remain almost unchanged in the presence of both pCFRC and rCF, while both types of composites see a reduction in the degree of crystallisation or crystal size, probably due to enhanced wPP degradation when it is melt-mixed with the fillers. Rheological studies have shown an increase in complex viscosity, and of both storage and loss moduli, in the presence of pCFRCs. This filler seems to interact more strongly with the wPP matrix than rCF. This can be due to a combined synergic effect of the presence of carbon fibre and epoxy resin fragments and/or the presence of intact carbon fibre sizing, which was likely removed or swollen during the epoxy matrix digestion process to reclaim the fibres. The presence of randomly oriented short rCFs increases the melt viscosity at low frequencies, until the fibres are aligned under the shear forces. Both types of fillers are evenly dispersed in the solid matrix of wPP, showing good adhesion with the matrix. An increase in the stiffness of the composites, without a significant reduction in tensile strength, is observed. Young’s modulus of wPP/pCFRC 20%wt is a little less than twice that of wPP, while for wPP/rCF 20%wt it increases more than three times. Conversely, as expected, the elongation at break is reduced by the presence of rigid fibres.

In conclusion, the results obtained demonstrate that two types of industrial waste can be successfully combined to be converted into sustainable composites that can be processed by compression and/or injection moulding and used to find new applications, thus contributing to waste reduction and resource efficiency in the polymer industry.

## Figures and Tables

**Table 1:** Sample composition and code.

Sample Code	wPP, %wt	pCFRC, %wt	rCF, %wt
wPP	100	-	-
wPP/pCFRC 1%wt	99	1	-
wPP/pCFRC 10%wt	90	10	-
wPP/pCFRC 20%wt	80	20	-
wPP/rCF 10%wt	90	-	10
wPP/pCF 20%wt	80	-	20

**Table 2:** DSC data from the first and second heating and the first cooling of wPP, wPP/pCFRC and wPP/rCF composites. Enthalpies are normalised with respect to the wPP matrix weight.

Sample	1st Heating		1st Cooling		2nd Heating	
	T (°C)	ΔH (J/g)	T (°C)	ΔH (J/g)	T (°C)	ΔH (J/g)
wPP	T_1_ ≈ 130				T_1_ ≈ 128	
	T_2_ ≈ 168	ΔH_2_ ≈ 93.1	T_1_ ≈ 125	ΔH_1_ ≈ −93.0	T_2_ ≈ 164	ΔH_2_ ≈ 93.1
wPP/pCFRC 1%wt	T_1_ ≈ 129				T_1_ ≈ 128	
	T_2_ ≈ 165	ΔH_2_ ≈ 75.1	T_1_ ≈ 126	ΔH_1_ ≈ −82.7	T_2_ ≈ 165	ΔH_2_ ≈ 76.4
wPP/pCFRC 10%wt	T_1_ ≈ 128				T_1_ ≈ 128	
	T_2_ ≈ 167	ΔH_2_ ≈ 75.7	T_1_ ≈ 125	ΔH_1_ ≈ −85.6	T_2_ ≈ 164	ΔH_2_ ≈ 79.9
wPP/pCFRC 20%wt	T_1_ ≈ 129				T_1_ ≈ 128	
	T_2_ ≈ 167	ΔH_2_ ≈ 85.8	T_1_ ≈ 124	ΔH_1_ ≈ −78.4	T_2_ ≈ 164	ΔH_2_ ≈ 81.3
wPP/rCF 10%wt	T_1_ ≈ 128				T_1_ ≈ 128	
	T_2_ ≈ 165	ΔH_2_ ≈ 76.9	T_1_ ≈ 125	ΔH_1_ ≈ −78.7	T_2_ ≈ 164	ΔH_2_ ≈ 81.6
wPP/rCF 20%wt	T_1_ ≈ 128				T_1_ ≈ 128	
	T_2_ ≈ 165	ΔH_2_ ≈ 77.6	T_1_ ≈ 125	ΔH_1_ ≈ −83.8	T_2_ ≈ 164	ΔH_2_ ≈ 83.5

**Table 3:** TGA results for wPP and its composites with CFRC and rCF.

Sample	T_onset_ (°C)	T_@50% weight loss_ (°C)	T_offset_ (°C)	Residual Mass_@600°C_ (%)
wPP	381	444	471	2.3
wPP/pCFRC 1%wt	307	435	470	4.1
wPP/pCFRC 10%wt	321	434	469	12.4
wPP/pCFRC 20%wt	314	435	469	16.6
wPP/rCF 10%wt	310	437	472	11.2
wPP/rCF 20%wt	271	427	467	19

## Data Availability

All data are available.
